# Shining a spotlight on immunometabolism

**DOI:** 10.1038/s42003-020-01280-x

**Published:** 2020-10-06

**Authors:** Shuai Jiang

**Affiliations:** Department of Microbiology and Immunology, Louisiana State University Health Sciences Center, Shreveport, LA 71130 USA

## Abstract

The metabolism of immune cells is a rapidly developing field with therapeutic implications. In recognition of this exciting area, our journal is welcoming submissions of primary research articles, perspectives, comments, and review articles in immunometabolism with the aim to highlight these articles in a special collection.

Metabolism has recently become the hub connecting several rapidly growing research areas, including cancer, hematopoietic stem cell biology, and cellular development^1–3^. Indeed, there is currently a keen interest in cellular reprogramming of different immune cell subpopulations and in understanding the functional role of metabolic factors—key enzymes, metabolites and nutrient transporters—in various immune cell subsets in our biology community. These exciting topics are commonly described under the term immunometabolism.

We at *Communications Biology* are welcoming submissions on immunometabolism as part of a special collection to be published in 2021.

One of the most exciting areas in immunometabolism is regarding memory immune cell subpopulations (e.g., germinal center independent memory B cells, T-bet memory B cells, central memory T cells, and tissue resident memory T cells), which are thought to exert their functional roles by adjusting diverse metabolic programs. Further studies are warranted to dissect the functional roles and molecular mechanisms of metabolites and enzymes of the TCA cycle and glycolysis within each memory immune cell subset. In addition, new insights into metabolism in immune-mediated pathological diseases, such as atherosclerosis, would advance the development of the immune metabolic biomarkers and potential clinical applications in cardiovascular medicine.

Recent evidence indicates that specifically targeting host metabolic reprogramming might provide the host with defenses to fight environmental challenges such as pathogen infections. For instance, targeting glutaminolysis has been suggested to regulate proinflammatory cytokine production and immune cell infiltration upon infection^4^. What are the molecular mechanisms underpinning immune-mediated pathologies? Can we target specific metabolic pathways in various immune cell subsets to clinically halt disease? These issues are worthy aims for future research. Uncovering the answers to all these questions might guide the development of immunometabolism-based therapies in future. As I know from my laboratory research activities on microRNAs in immunometabolism, further studies of metabolic alterations by microRNAs in the context of different immune cell subsets are a potential hot topic in this exciting area.

## Call for papers in immunometabolism

High-quality articles in the areas immunology and metabolism have already found their home in *Communications Biology* since it was launched in early 2018. We have been serving as a harbor for immunologists, endocrinologists and scientists who study metabolism. As illustrated by our journal’s name, communication between biologists is what we envisioned to accomplish when we launched our journal. Following this in our heart, our in-house editors and external editorial board members will keep working hard to provide a home for scientists who advance this exciting area by publishing new findings that are relevant to the field.

We at *Communications Biology*^5^ are welcoming submissions on immunometabolism as part of a special collection to be published in 2021. We especially encourage submissions related to metabolic enzymes in innate/adaptive immune responses, nutrient transporters in immune cell activation, metabolites as immunological regulators, strategies targeting metabolism to modulate immune responses, and non-coding RNAs in regulation of immunometabolism; however, we will also consider other areas of immunometabolism. Papers will be published as they are ready and combined into a special collection once a majority have been published. We will consider all submissions related to immunometabolism submitted by March 15, 2021 for the collection. We hope this special collection will benefit scientists who are interested in the state-of-the-art research on immune responses and cellular metabolic reprogramming.
